# Supplementary material for: Adenosine A2A receptor agonist polydeoxyribonucleotide ameliorates short-term memory impairment by suppressing cerebral ischemia-induced inflammation via MAPK pathway
Source: PLoS One. 2021 Mar 18;16(3):e0248689. doi: 10.1371/journal.pone.0248689 (PMC7971468; doi:10.1371/journal.pone.0248689)
Supplement: S1 File — (DOCX) [file pone.0248689.s001.docx]

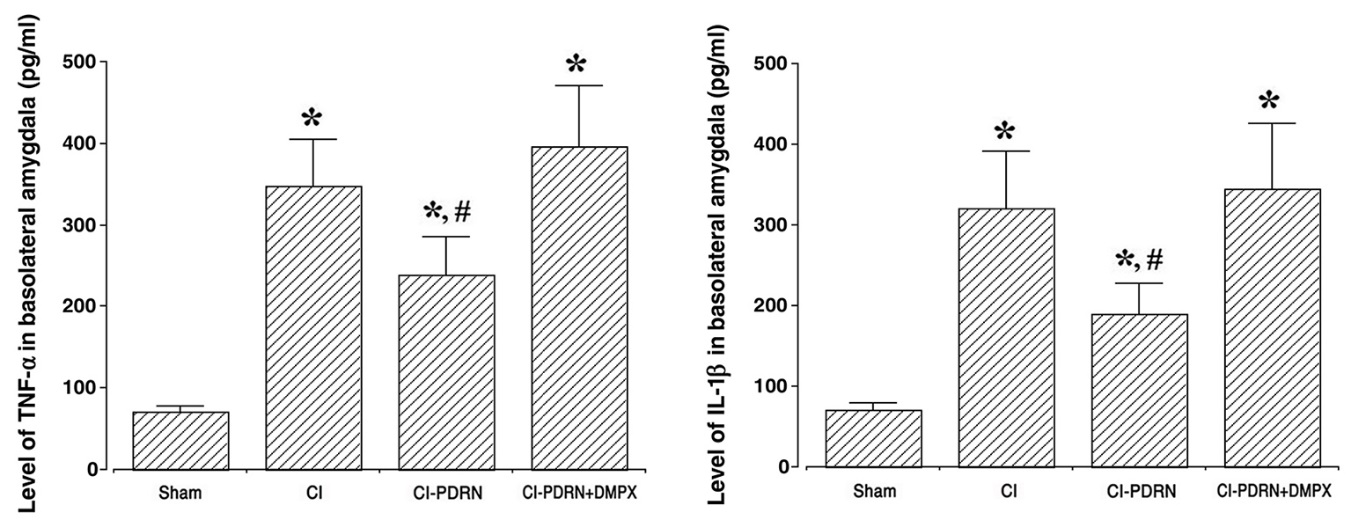


**Supplement 1. Altered expression of pro-inflammatory cytokines in the basolateral amygdala.** Left. Concentration of tumor necrosis factor-α (TNF-α) in basolateral amygdala. Right. Concentration of interleukin (IL)-1β in basolateral amygdala. Sham, sham-operation group; CI, cerebral ischemia-induced group; CI-PDRN, cerebral ischemia-induced and polydeoxyribonucleotide (PDRN)-treated group; CI-PDRN+DMPX, cerebral ischemia-induced and PDRN with 7-dimethyl-1-propargylxanthine (DMPX)-treated group. * indicates P < 0.05 compared with the sham-operation group. # indicates P < 0.05 compared with the cerebral ischemia-induced group.


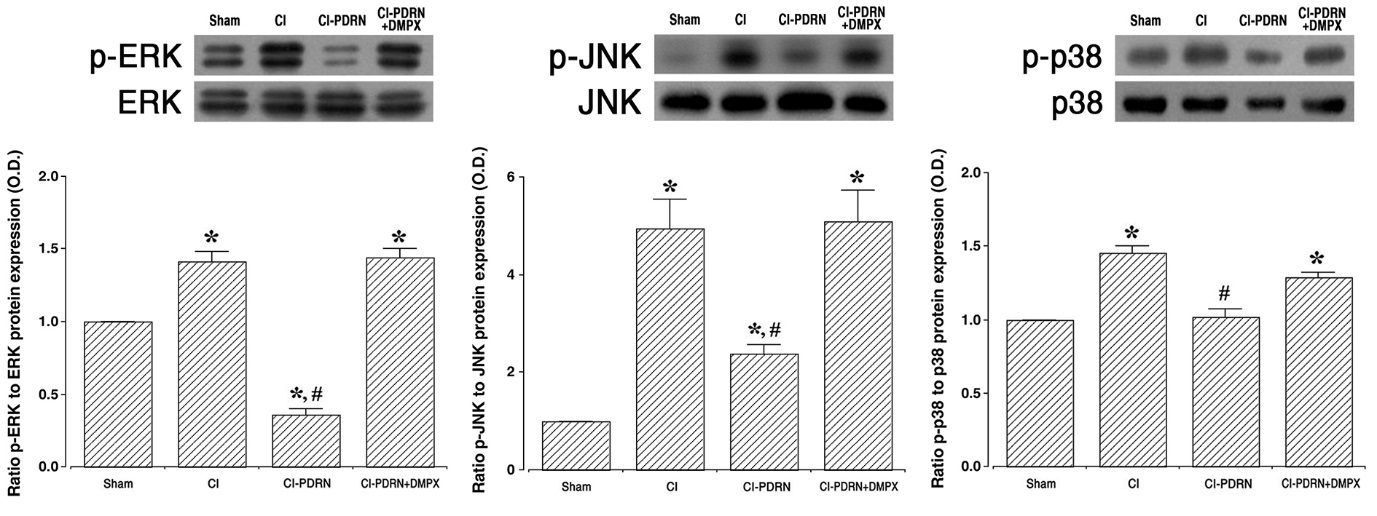


**Supplement 2. Changes in the mitogen-activated protein kinase (MAPK) cascade in the basolateral amygdala.** Left. Ratio of phosphorylated extracellular signal-regulated kinases (p-ERK) to ERK. Middle. Ratio of phosphorylated c-Jun NH_2_-terminal kinases (p-JNK) to JNK. Right. Ratio of phosphorylated p38 kinase (p-p38) to p38 in the basolateral amygdala. Sham, sham-operation group; CI, cerebral ischemia-induced group; CI-PDRN, cerebral ischemia-induced and polydeoxyribonucleotide (PDRN)-treated group; CI-PDRN+DMPX, cerebral ischemia-induced and PDRN with 7-dimethyl-1-propargylxanthine (DMPX)-treated group. * indicates P < 0.05 compared with the sham-operation group. # indicates P < 0.05 compared with the cerebral ischemia-induced group.


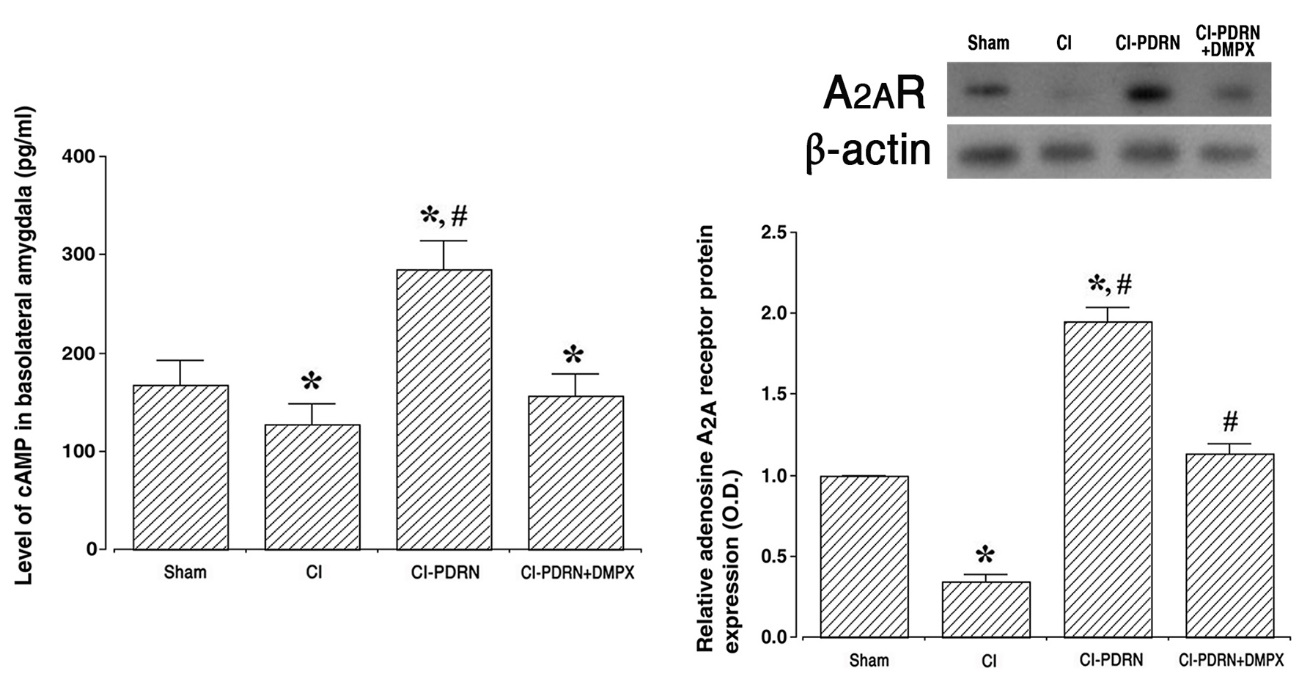


**Supplement 3. Changes in cAMP concentration and adenosine A_2A_ receptor expression in basolateral amygdala.** Left. Concentration of cAMP in basolateral amygdala. Right. The relative expression of the adenosine A_2A_ receptor in the basolateral amygdala. Sham, sham-operation group; CI, cerebral ischemia-induced group; CI-PDRN, cerebral ischemia-induced and polydeoxyribonucleotide (PDRN)-treated group; CI-PDRN+DMPX, cerebral ischemia-induced and PDRN with 7-dimethyl-1-propargylxanthine (DMPX)-treated group. * indicates P < 0.05 compared with the sham-operation group. # indicates P < 0.05 compared with the cerebral ischemia-induced group.


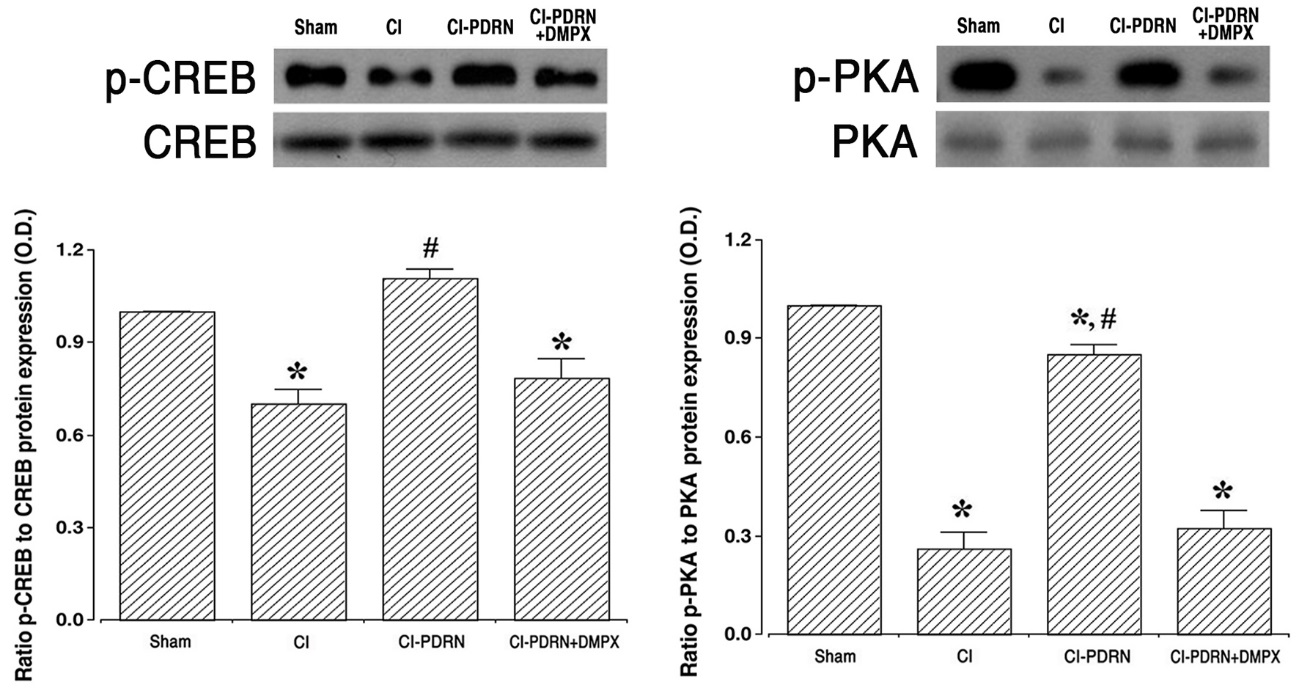


**Supplement 4. Changes in phosphorylated cAMP response element-binding protein (p-CREB) to CREB ratio and phosphorylated protein kinases A (p-PKA) to PKA ratio in the basolateral amygdala.** Left. Ratio of p-CREB to CREB in the basolateral amygdala. Right. Ratio of p-PKA to PKA in the basolateral amygdala. Sham, Sham-operation group; CI, cerebral ischemia-induced group; CI-PDRN, cerebral ischemia-induced and polydeoxyribonucleotide (PDRN)-treated group; CI-PDRN+DMPX, cerebral ischemia-induced and PDRN with 7-dimethyl-1-propargylxanthine (DMPX)-treated group. * indicates P < 0.05 compared with the sham-operation group. # indicates P < 0.05 compared with the cerebral ischemia-induced groups.
