# Supplementary figures and images for: Adenosine A2A receptor agonist polydeoxyribonucleotide ameliorates short-term memory impairment by suppressing cerebral ischemia-induced inflammation via MAPK pathway
Source: PLoS One. 2021 Mar 18;16(3):e0248689. doi: 10.1371/journal.pone.0248689 (PMC7971468; doi:10.1371/journal.pone.0248689)

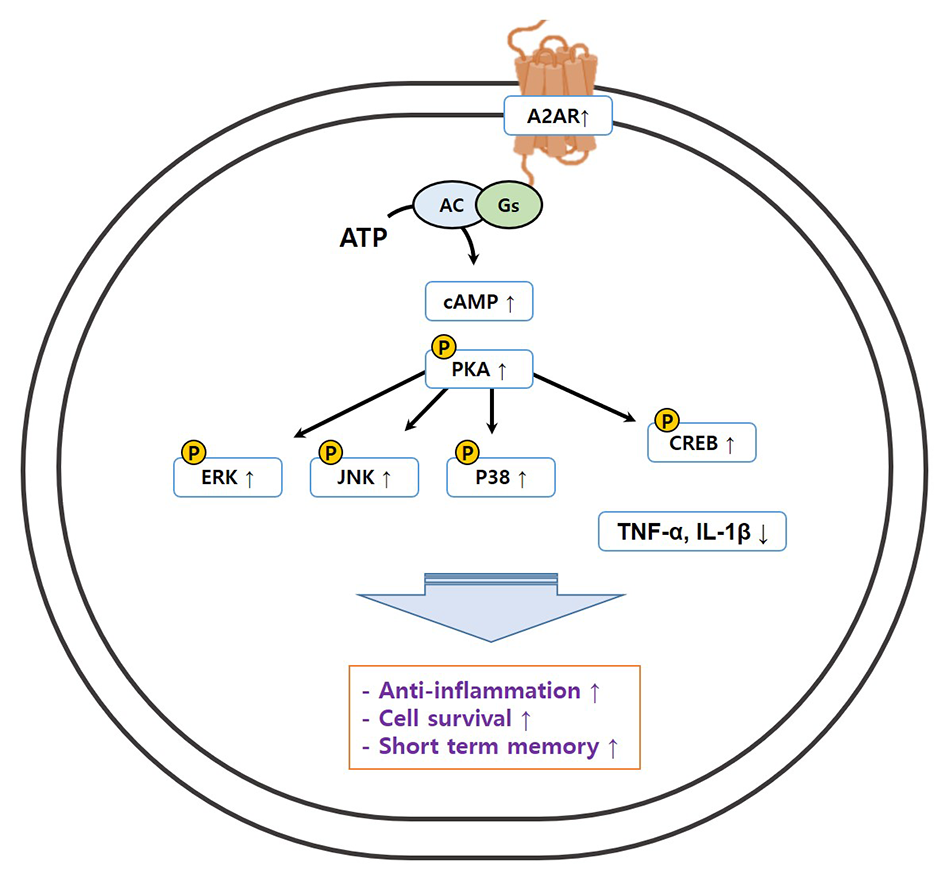

Supplement: S1 Graphic abstract — (TIF) [file pone.0248689.s002.tif]
