# Supplementary material for: Adenosine A2A receptor agonist polydeoxyribonucleotide ameliorates short-term memory impairment by suppressing cerebral ischemia-induced inflammation via MAPK pathway
Source: PLoS One. 2021 Mar 18;16(3):e0248689. doi: 10.1371/journal.pone.0248689 (PMC7971468; doi:10.1371/journal.pone.0248689)

[Western blot raw band image-Manuscript’s main fiugres]-1

Figure 4

→
Sham CI CI-PDRN CI-PDRN+DMPX

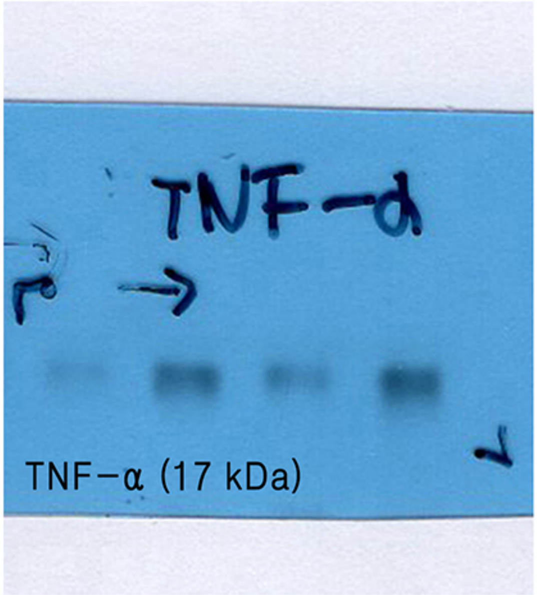

→
Sham CI CI-PDRN CI-PDRN+DMPX

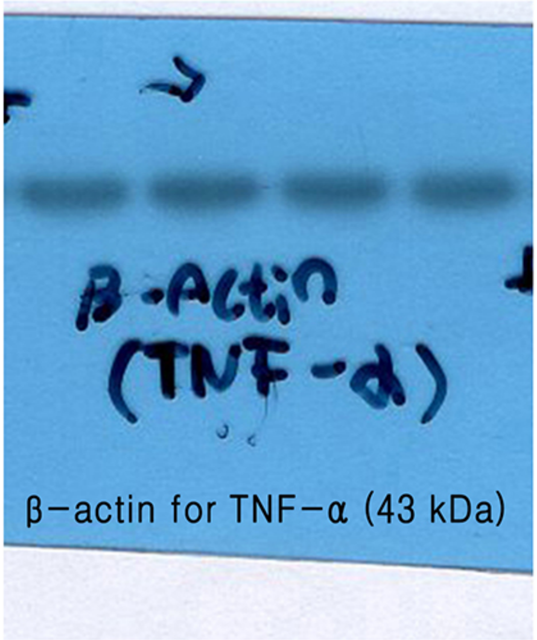

→
Sham CI CI-PDRN CI-PDRN+DMPX

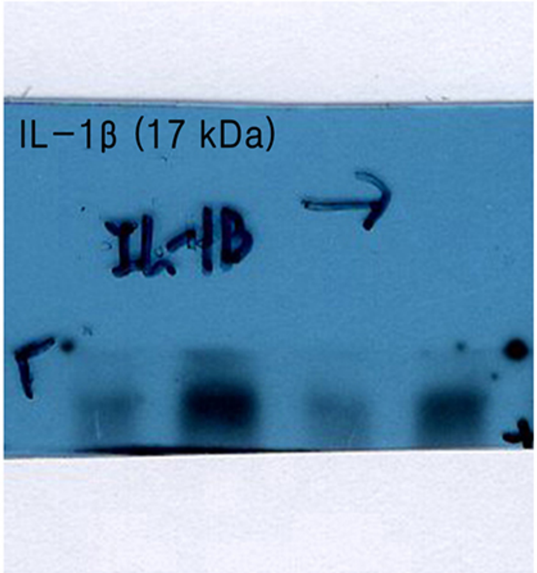

→
Sham CI CI-PDRN CI-PDRN+DMPX

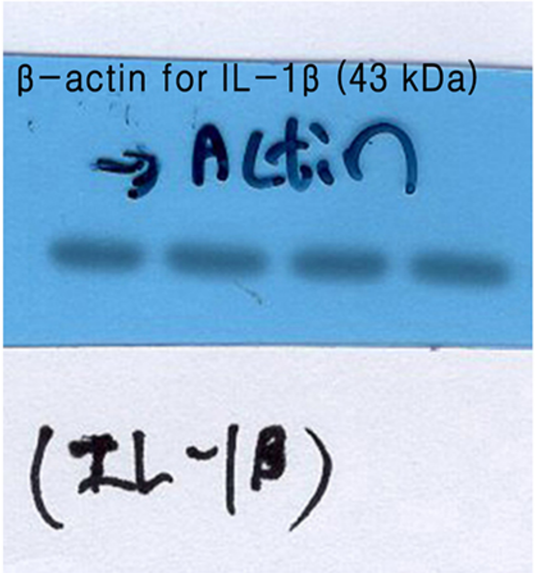

[Western blot raw band image-Manuscript's main fiugres]-2

Figure 5

→ Sham CI CI-PDRN CI-PDRN+DMPX

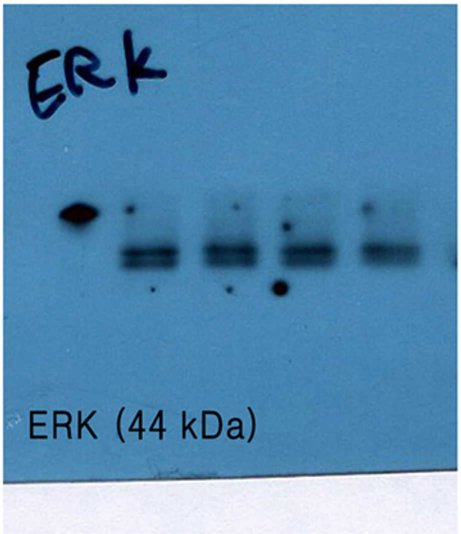

→ Sham CI CI-PDRN CI-PDRN+DMPX

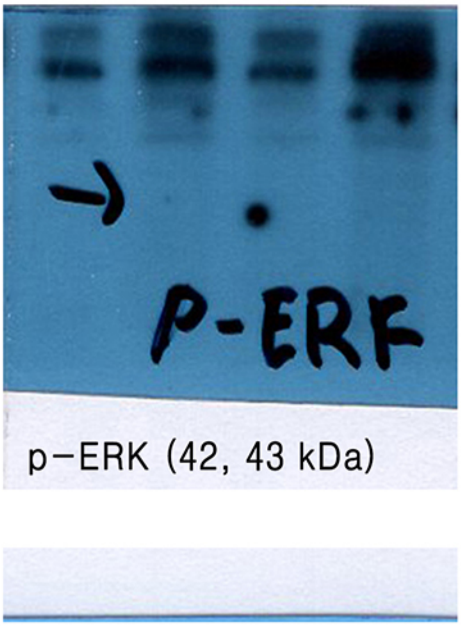

→ Sham CI CI-PDRN CI-PDRN+DMPX

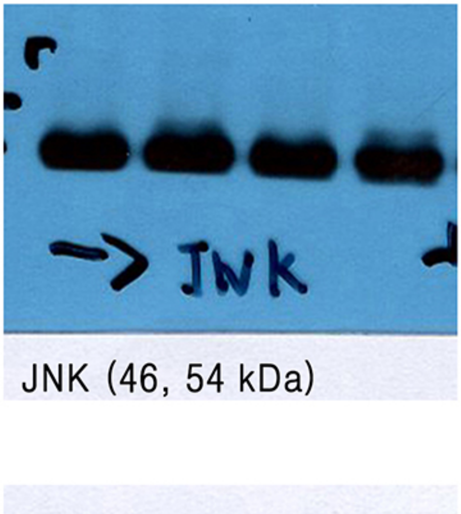

→ Sham CI CI-PDRN CI-PDRN+DMPX

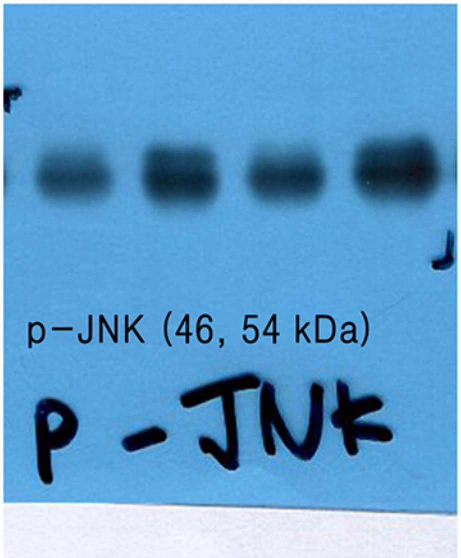

CI-PDRN+DMPX CI-PDRN CI Sham ←

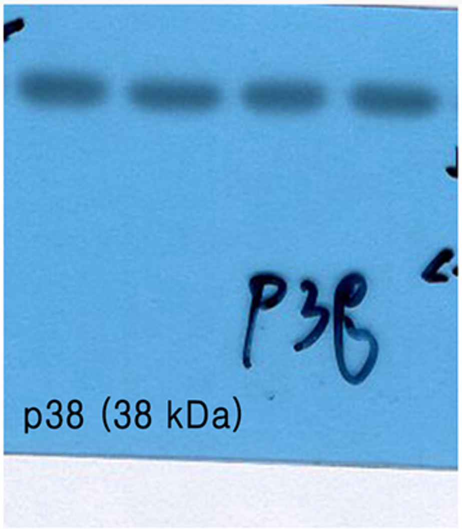

→ Sham CI CI-PDRN CI-PDRN+DMPX

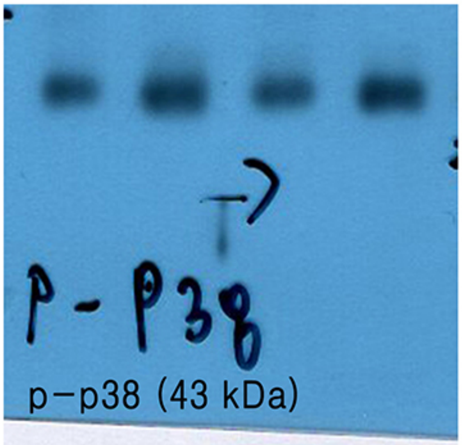

[Western blot raw band image-Manuscript’s main fiugres]-3

Figure 6

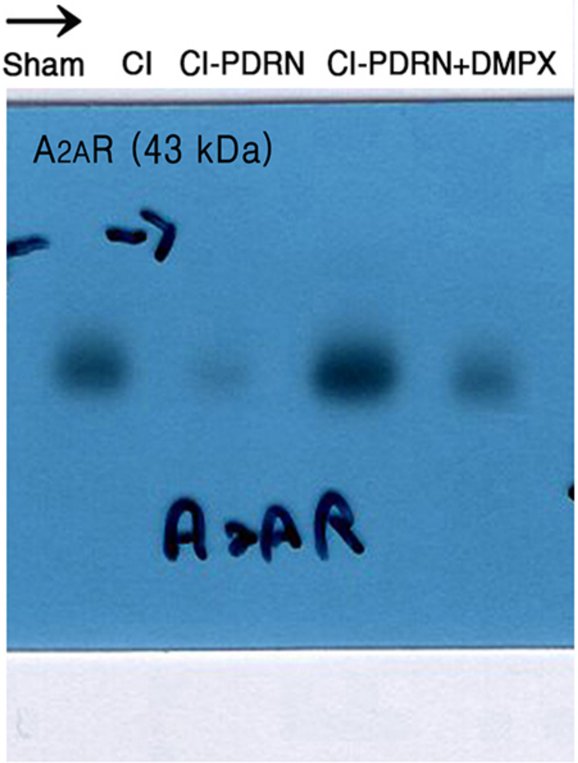

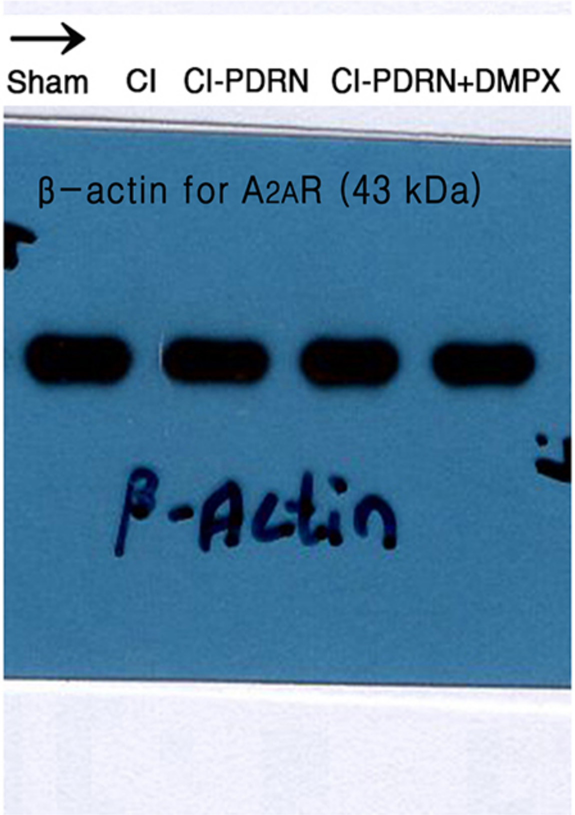

[Western blot raw band image-Manuscript's main fiugres]-4

Figure 7

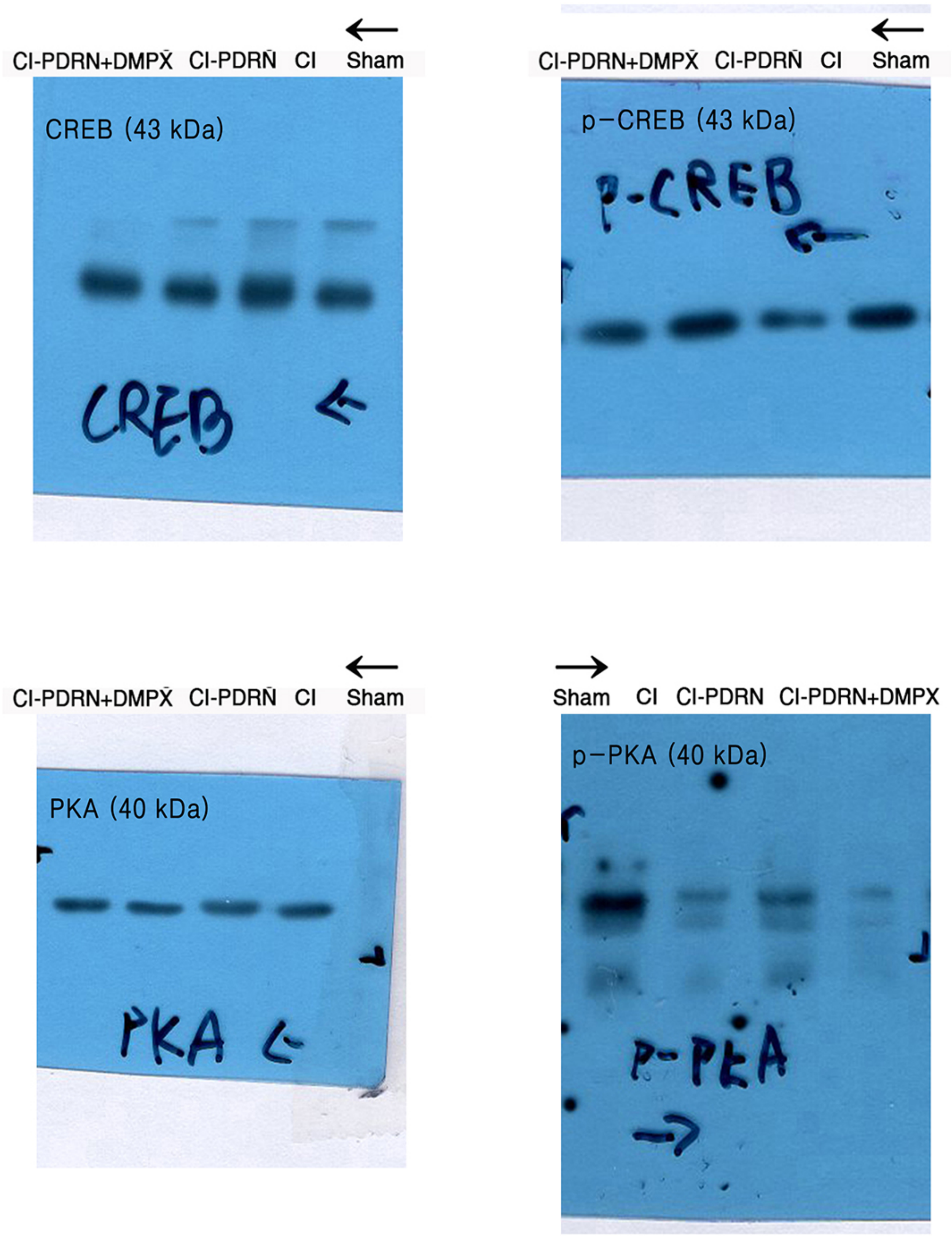

[Western blot raw band image-Supplemental fiugres]-1

Supplement 2

→

Sham CI CI-PDRN CI-PDRN+DMPX

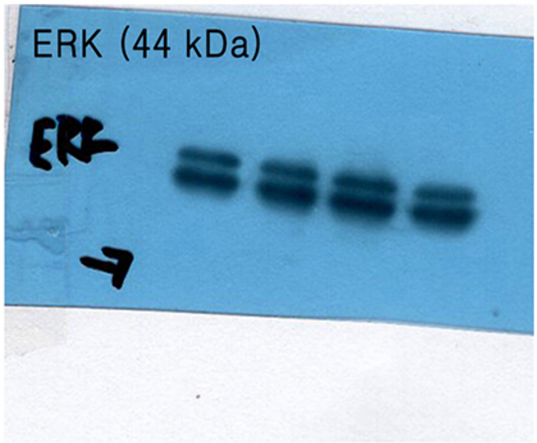

→

Sham CI CI-PDRN CI-PDRN+DMPX

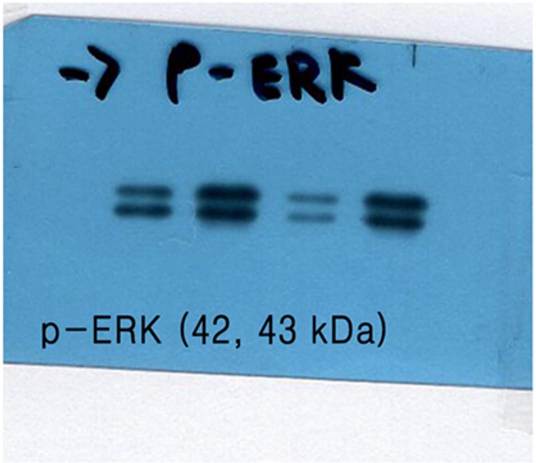

←

CI-PDRN+DMPX CI-PDRN CI Sham

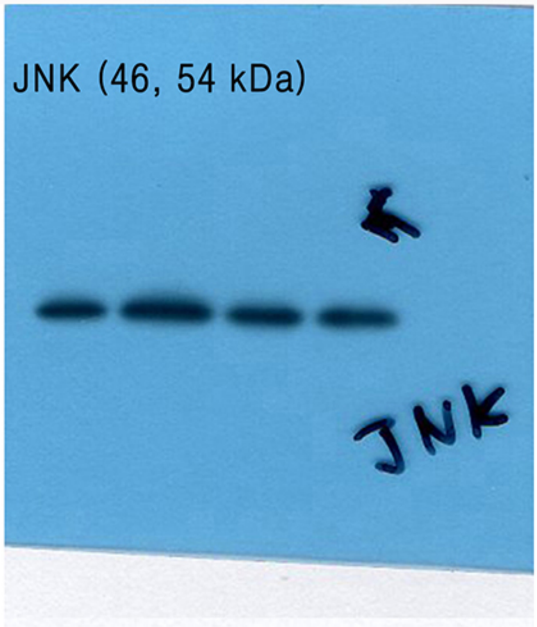

→

Sham CI CI-PDRN CI-PDRN+DMPX

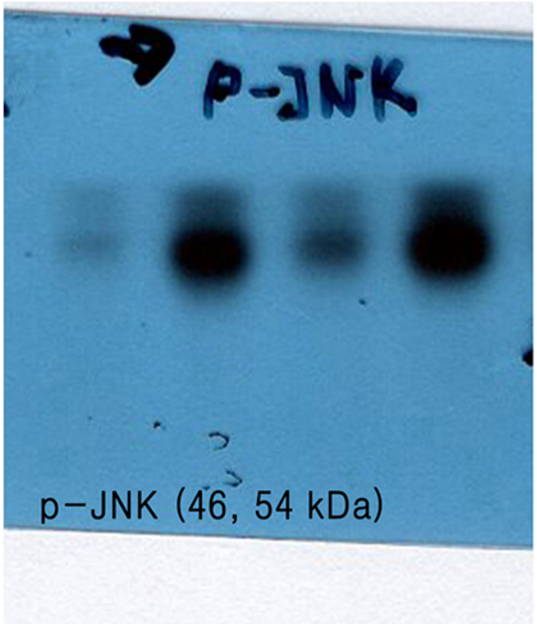

→

Sham CI CI-PDRN CI-PDRN+DMPX

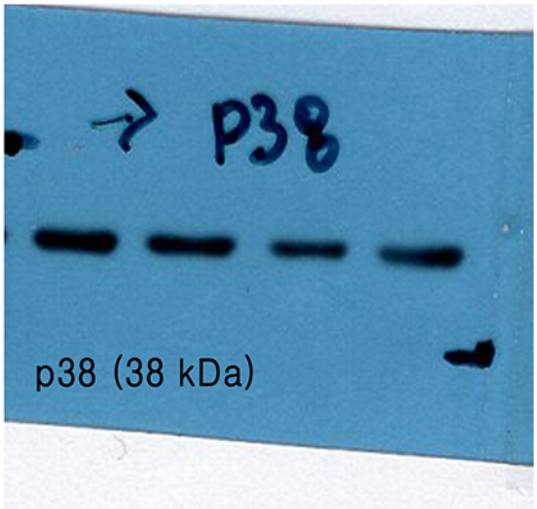

←

CI-PDRN+DMPX CI-PDRN CI Sham

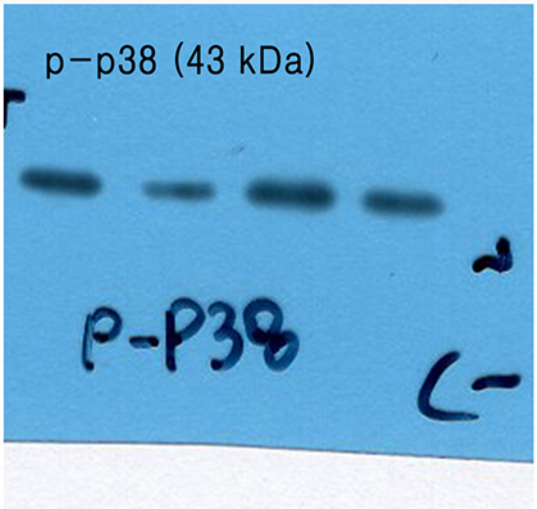

[Western blot raw band image-Supplemental fiugres]-2

Supplement 3

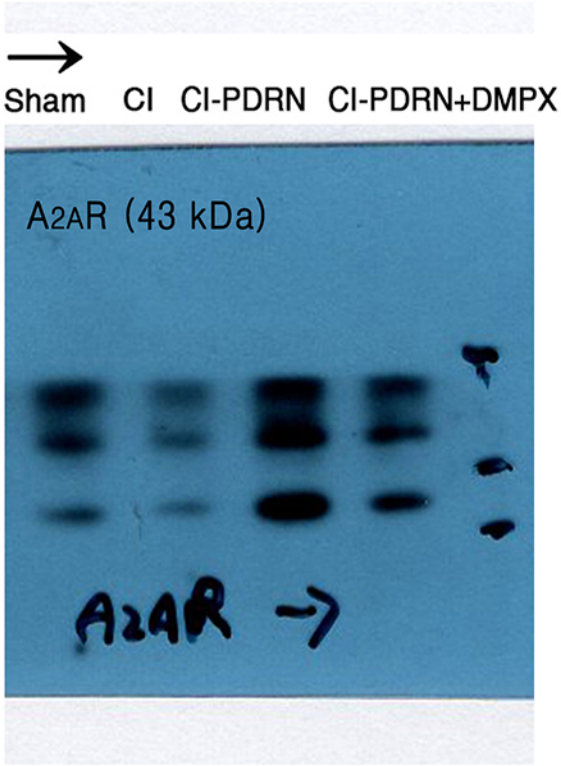

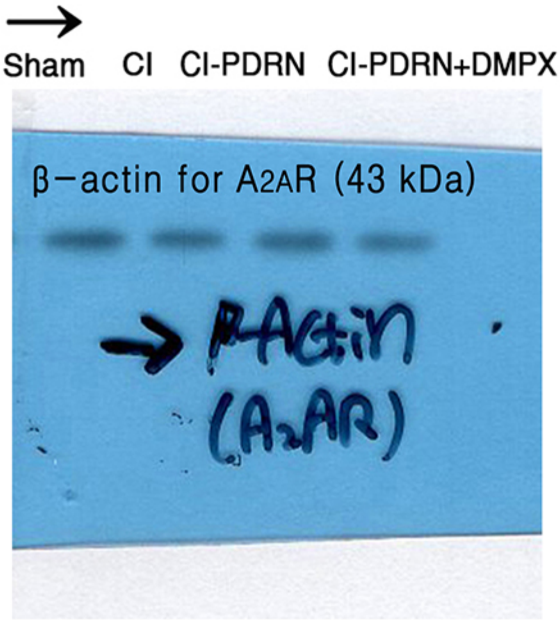

[Western blot raw band image-Supplemental fiugres]-3

Supplement 4

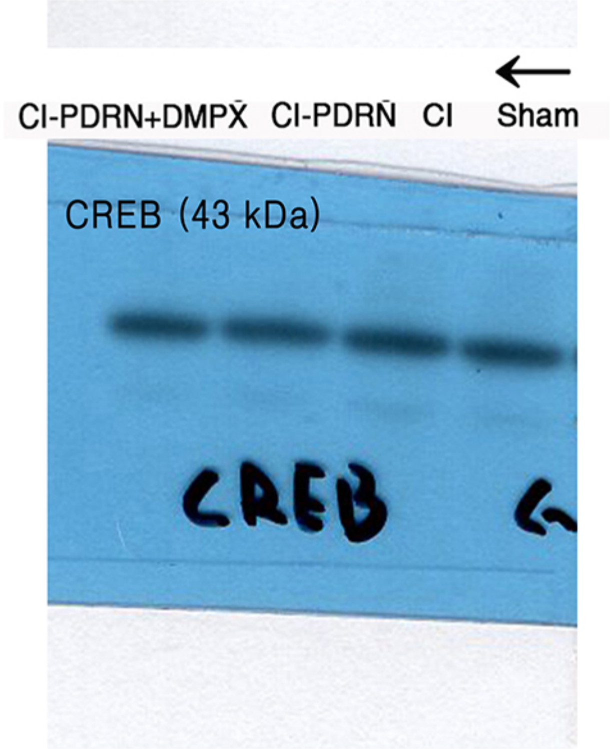

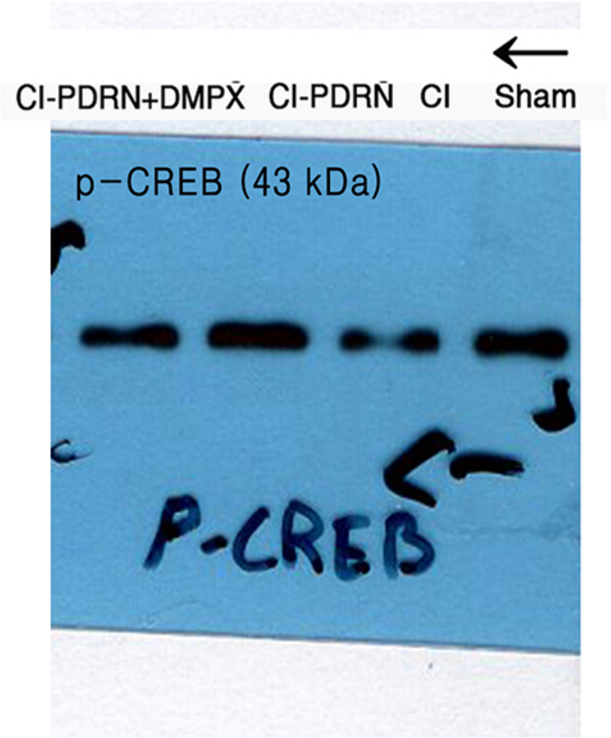

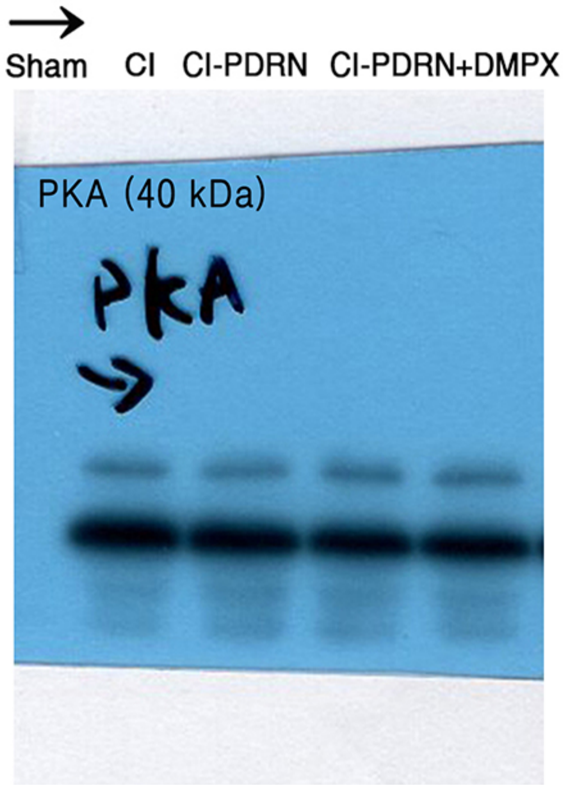

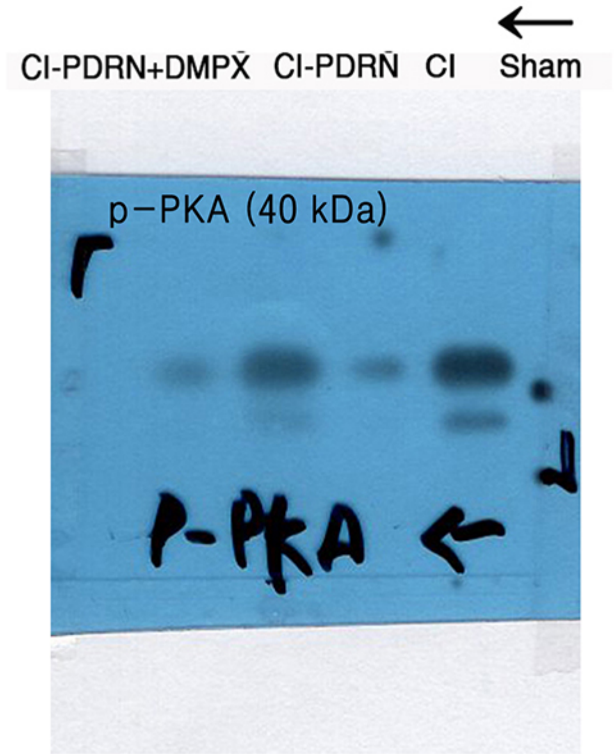

Supplement: S1 Raw images — (PDF) [file pone.0248689.s003.pdf]
