# Supplementary material for: Adenosine A2A receptor agonist polydeoxyribonucleotide ameliorates short-term memory impairment by suppressing cerebral ischemia-induced inflammation via MAPK pathway
Source: PLoS One. 2021 Mar 18;16(3):e0248689. doi: 10.1371/journal.pone.0248689 (PMC7971468; doi:10.1371/journal.pone.0248689)
Supplement: Attachment — Submitted filename: Response to Reviewers.docx [file pone.0248689.s005.docx]

**Answers to Reviewers’ Comments**

**Manuscript ID:** PONE-D-20-25445

**Title:** Adenosine A_2A_ receptor agonist polydeoxyribonucleotide ameliorates short-term memory impairment by suppressing cerebral ischemia-induced inflammation via MAPK pathway

Authors: Il-Gyu Ko, Jun-Jang Jin, Lakkyong Hwang, Sang-Hoon Kim, Chang-Ju Kim, Jung Won Jeon, Jun-Young Chung, Jin Hee Han

We sincerely appreciate for your kind advice and comments to our manuscript. We revised the manuscript according to the reviewer’s comments. We added new experimental data, and modifications were expressed in red.

**Reviewer #1**

The manuscript is in line with the previous comments. The abstract lacks a reference to the brain areas where the measurements were made.

**Answer:** According to reviewer comment, we have added word in the abstract.

**• Following word was added into the Abstract.**

In the current study, induction of ischemia enhanced the levels of pro-inflammatory cytokines and increased phosphorylation of MAPK signaling factors in the hippocampus and basolateral amygdala.

**Reviewer #2**

The present paper by Il-Gyo Ko and collegues aims to demonstrate the therapeutic effect of PDRN (polydeoxyribonucleotide), an adenosine A_2A_ receptor agonist, on cerebral ischemia in gerbils by suppressing the secretion of pro-inflammatory cytokines with an anti-inflammatory effect. This action is accompanied by increased phosphorylation of MAPK in hippocampus and an amelioration in short-term memory in gerbils. The model of global cerebral ischemia performed is of 2-common carotid arteries occlusion for 7 minutes.

The manuscript is not original, since there is a lot of evidence about the neuroprotective role for PDRN in cerebral ischemia in rodents. At the same time, it has been widely demonstrated the involvement of A2A adenosine receptors in cerebral ischemia reperfusion injury, in the signaling to phosphorylated extracellular signal-regulated protein kinase (pERK1/2). The survival pathway evoked is clearly linked to an amelioration in short-term memory performances in rodent models of global cerebral ischemia, where CA1 hippocampal populations are mainly impaired. Therefore, the paper is not innovative, not suitable of publication on PLOS journal.

**Answer:** A search for "PDRN" and "ischemia" in PubMed only shows 6 results: renal ischemia, skin ischemia, testicular ischemia, and peripheral arterial occlusion. There have been no studies on the effect of PDRN on the central nervous system, particularly the brain. Therefore, this study is considered to be the first to evaluate the action and mechanism of PDRN on brain ischemia. In this study, we first identified the pharmacological action of PDRN in brain ischemia, and proved that this paper has sufficient originality.

We have demonstrated that PDRN treatment promoted the signaling of the cAMP-PKA pathway by activating the adenosine A_2A_ receptor and enhanced phosphorylation of CREB. It was also found that increased cAMP concentration reduced the expression of pro-inflammatory cytokines by inhibiting the phosphorylation level of the MAPK cascade pathway. This mechanism of action of PDRN has been proven to be effective in ameliorating short-term memory loss. In this paper, we identified the mechanism of action of PDRN during brain ischemia, and contained very innovative contents.

In addition, the PDRN used in this study is one of many adenosine A_2A_ receptor agonists, whose origin differs from conventional drugs. PDRN (source: DNA extracted from salmon sperm cells) used in this study has very high stability and pharmacological action unlike compounds extracted from mammalian organs or PDRN (eg Defibrotide). Thus, this study evaluated the therapeutic efficacy of cerebral ischemia using PDRN, a highly stable drug. In this paper, we deal with PDRN, a very stable drug, and contain novel discoveries.

Therefore, we disagree with the reviewers' opinion that our work is not innovative. We think this paper has originality containing innovative contents and novel discoveries.

**Answer to Journal Comments**

**Manuscript ID:** PONE-D-20-25445

**Title:** Adenosine A_2A_ receptor agonist polydeoxyribonucleotide ameliorates short-term memory impairment by suppressing cerebral ischemia-induced inflammation via MAPK pathway

Authors: Il-Gyu Ko, Jun-Jang Jin, Lakkyong Hwang, Sang-Hoon Kim, Chang-Ju Kim, Jung Won Jeon, Jun-Young Chung, Jin Hee Han

**Q1.** Please include a legend for figure 'Graphic abstract'.

**Answer:** According to the journal’s comments, following words was inserted into “Graphic Abstract” in the Figure legends part.

**• Following word was added into the Figure legends.**

**Graphic Abstract**

**Q2.** Please ensure that you refer to Figure 'Graphic abstract' in your text as, if accepted, production will need this reference to link the reader to the figure.

**Answer:** According to the journal’s comments, following words was inserted into “graphic abstract” in the manuscript.

**• Following word was added into the manuscript.**

The current study has revealed that PDRN treatment inhibits inflammation, improves neuronal cell survival, and prevents a decline in short-term memory in a brain ischemia animal model (graphic abstract).
